# The motor development of orphaned children with and without HIV: Pilot exploration of foster care and residential placement

**DOI:** 10.1186/1471-2431-11-11

**Published:** 2011-02-07

**Authors:** Jennifer Jelsma, Nailah Davids, Gillian Ferguson

**Affiliations:** 1 Division of Physiotherapy, School of Health and Rehabilitation Sciences, Faculty of Health Sciences, University of Cape Town, Anzio Road, Observatory 7925, South Africa

## Abstract

**Background:**

The AIDS epidemic has lead to an increase in orphaned children who need residential care. It is known that HIV leads to delayed motor development. However, the impact of place of residence on motor function has not been investigated in the South African context. The aim of the study was therefore to establish if children in institutionalised settings performed better or worse in terms of gross motor function than their counterparts in foster care. A secondary objective was to compare the performance of children with HIV in these two settings with those of children who were HIV negative.

**Methods:**

Forty-four children both with and without HIV, were recruited from institutions and foster care families in Cape Town. The Peabody Development Motor Scale (PDMS II) was used to calculate the total motor quotient (TMQ) at baseline and six months later. Comparisons of TMQ were made between residential settings and between children with and without HIV.

**Results:**

Twenty-one children were infected with HIV and were significantly delayed compared to their healthy counterparts. Antiretroviral therapy was well managed among the group but did not appear to result in restoration of TMQ to normal over the study period. HIV status and place of residence emerged as a predictor of TMQ with children in residential care performing better than their counterparts in foster care. All children showed improvement over the six months of study.

**Conclusions:**

Foster parents were well supported administratively in the community by social welfare services but their children might have lacked stimulation in comparison to those in institutional settings. This could have been due to a lack of resources and knowledge regarding child development. The assumption that foster homes provide a better alternative to institutions may not be correct in a resource poor community and needs to be examined further.

## Background

South Africa has been severely affected by the HIV pandemic. In 2005 there were an estimated 2.5 million orphans [1], a number expected to grow to 4 million by 2015 (10% of South African population). It is anticipated that the extended family system will find it increasingly difficult to absorb these orphans due to continued severe economic constraints [2]. Therefore a need exists for appropriate placement and various types of care settings have been established in different communities [3]. These include foster care, institutionalisation and adoption. In South Africa it is estimated that about 29 000 children are cared for in 169 registered children's homes and 37 places of safety and many residential homes have now opened their doors to house children affected and infected by HIV/AIDS [4].

Despite the magnitude of the problem, little evidence exists to support the effectiveness of different interventions intended to improve the quality of life of these orphaned and vulnerable children [5]. Although it is generally accepted that foster care is the preferred option, the effect of institutionalization is controversial [6]. Some authors maintain that institutions contribute to a large extent to global developmental delay of the children they house [7] and may be unable to provide the individualized nurturing found in loving and responsible families and households [8]. The caregiver to child ratio, which varies across institutions, may also be limited and thus contribute to a delay in emotional and behavioural development [9].

However, foster care may also not allow for optimal development. Changes in foster care placements, exposure to alcoholism, drug abuse, possible neglect and abandonment are all factors which may impact on the physical, emotional and behavioural development of these children with the subsequent interference with learning [2 10]. There is an acknowledgement that "in some cases of extremely adverse rearing circumstances well-functioning child-care institutions may offer children a better rearing environment than their own dysfunctional families" p237 [6].

The influence of HIV on development has been well documented. HIV infects the developing Central Nervous System (CNS) of children and the virus is known to enter the CNS early in the course of the disease [11,12]. Both motor and cognitive development are affected and delay may be present despite the initiation of anti-retroviral therapy (ARVT). Even without the influence of opportunistic infections, motor performance has been found to be about 75% of the level of typically developing children [13].

The aim of the study was therefore to explore the impact of place of residence on the motor development of children who were infected with HIV. This was done by comparing the motor development of four groups of children aged 3-6 years: those with HIV/AIDS residing in institutions, those with HIV/AIDS cared for by foster parents, children without HIV residing in institutions and children without HIV living with foster parents. using the Peabody Developmental Motor Scale (PDMS II) [14]. The change in motor developmental quotient over time was also investigated.

## Methods

A descriptive, prospective, analytical repeated measure study design was used. Data collection took place from July 2006 to June 2007. A sample of convenience was identified across all groups identified as part of the study. Participants were selected through foster care agencies and institutions in three suburbs in the Western Cape. Cross-sectional data was compared between the participant groups at base-line and six months later.

### Research Setting

Orphaned or abandoned children in South Africa may be placed either in an institution or with foster parents if their natural parents are unable or unwilling to care for them. The Children's Court is the guardian of all children and there are specific guidelines set out by the Department of Social Development i.e. National Guidelines for Social Services for children infected and affected by HIV/AIDS [3]. Included in this document are guidelines that pertain to the placement of orphaned or abandoned children in foster care, a place of safety or an institution. Once a child's home circumstances are investigated, and a child is thought to be in need of care, the social welfare officer must investigate options for placement which may include care in an institutional setting. Alternatively, if there is interest to foster a child, a Social Worker investigates the home and family circumstances of the foster parent/s and a court order allows the interested parties to care for the child.

There were three institutions included in the study with the number of residents ranging from to 30 to 120. Children lived in dormitories where two carers attended to 12 to 20 children. Two institutions had facilities for formal pre-school activities. All institutions had a large number of volunteers implementing activity programmes, music programmes and assistance with daily care of the children.

During the course of the study and independent of the study, the amount of stimulation afforded to the children in foster care was greatly increased. Social welfare officers and community field workers combined resources and provided foster care parents with emotional support and training on coping with a foster care child and a child infected with HIV/AIDS.

### Participants

Social welfare officers concerned with placement of children in foster homes were approached to assist in identification of children in the foster care group. Children with and without HIV were identified in each of the two settings, institutions and foster homes and in this manner the four groups listed above were identified. Children born with a neurological impairment or congenital abnormality were excluded after examination. In all children, three or more hospital admissions as reported by the caregiver or person in charge disqualified the child from participating as repeated hospitalization for whatever reason in itself might lead to developmental delay.

### Instrumentation

A questionnaire in English and isi-Xhosa was developed to determine the demographic, socio-economic and medical characteristics of participants. Weight and height was measured prior to test administration and the percentile for the child's age was calculated, using the Centers for Disease Control (....)norms [15].

The PDMS II is a normed test which assesses gross and fine motor skills of children 0-84 months of age [14]. It results in a fine motor quotient (FMQ), a gross motor quotient (GMQ) and a total motor quotient (TMQ). A small study found that children living in Cape Town, performed within the developmental range as normed on American children (Hartley Amien, personal communication) and it was assumed that the test was valid for the study subjects. Reliability of test administration was ensured through a pilot study on eight subjects who were similar in age to the participants. The inter-rater reliability was deemed adequate (GMQ - rho = .97, p < .01; FMQ rho = .95, p <.01 and TMQ rho = 1.00).

### Procedure

Approval for the study was granted by the University of Cape Town Medical Research Ethics Committee.

Fostered children were identified through the foster care agencies and appointments were arranged by the social workers along with the researcher. Informed consent was obtained from the appropriate authority (social welfare officers and foster parents) and permission granted by heads of the institutions. Testing was done in a quiet venue with care-givers present. Baseline testing was followed by a second test administration at six months.

### Data analysis

Analysis was done with STATISTICA Version 7. Descriptive statistics were used to present frequencies and demographic data. The Chi-Squared test was used to test for associations between gender, HIV status and place of residence. For numeric, normally distributed data, tested by using the Kolmorogov Smirnoff test, the independent t-test was used to determine if there was a significant difference in the mean scores of different groups. Forward stepwise multiple regression analysis was performed with TMQ as the dependent variable for baseline and six months. Age and BMI and dummy variables (coded as 0, 1) for gender, residence and HIV status were entered. Inclusion of an interaction term of residence*HIV status did not improve the predictive value (Adjusted R^2 ^= .415) and this was excluded from the final model.

## Results

A total of 61 children were recruited to participate in the study. All foster children had been placed into foster care by social workers working in conjunction with the community workers and had previously been in institutions or places of safety. There were 37 children in institutions and 24 children in foster care who were screened for eligibility. No foster parents refused to participate in the study. Seventeen participants were excluded due to meeting exclusion criteria after screening. A total of 44 children therefore entered into the study. All children were from areas that were under resourced and poor.

### Demographic details

The mean age of participants at baseline was 52.8 months (SD = 10.9; Range = 35.7 - 73.8 months). There was no difference in mean age between the samples (foster care 53.8, SD = 10.7; institutions 52.0, SD = 11.2 , p = .583), in the mean age at which children were placed in foster care (24 months, SD = 20.1) and in institutions (28.3, SD = 19.2, p = .5) or the mean time spent in foster care (31.4,SD = 18.8, range 4-58 months) or institutions (26.4, SD = 17.7, 1-65 months, p = .390).

Table 1 demonstrates the residence, gender and HIV status of the sample. There was no association between gender and residence (Chi = .15, p = .70) or between HIV status and residence (Chi = .002, p = .97). In summary, there appeared to be no difference in the foster care and institutionalised samples with regard to demographic variables.

**Table 1:** Status and residence

Residence	Gender	Status Tested negative	Status Tested positive	Totals
**Foster**	Female	6	2	8

**Foster**	Male	3	8	11

***Total Foster***		*9*	*10*	*19*

**Home**	Female	5	7	12

**Home**	Male	7	6	13

***Total Home***		*12*	*13*	*25*

***Total***		***21***	***23***	***44***

Status and residence of the complete sample (n = 44)

The mean age of foster mothers was 46.5 years (SD = 9.2, range 30-59). Eleven foster families lived in brick housing and eight lived in informal housing. All children who tested HIV positive were on antiretroviral therapy (ARVT) and all had been on treatment for five months or more at baseline with over half on treatment for 18 months or longer. Most children with HIV were reported to have experienced serious illness during the course of their lives and these are reported in Table 2.

**Table 2:** Opportunistic infections

	Count	Percent
**Gastro-enteritis**	14	60.9

**Tuberculosis**	13	56.5

**Ear Infection**	7	30.4

**Pneumonia**	5	21.7

**Seizure**	5	21.7

**Meningitis**	2	8.7

Frequency of opportunistic infections over the life-span of the HIV positive group (n = 23)

The two children who had had meningitis showed no obvious signs of motor dysfunction and were included in the study. Only one out of 44 children had been admitted to hospital in the three months prior to commencement of the study. She spent one day in hospital following seizures. During follow-up data collection, one child was placed in foster care and was admitted to hospital due to serious illness as a result of neglect. He was therefore not tested at follow-up, although his baseline data were included.

### Scores on PDMSII

**Figure 1:**
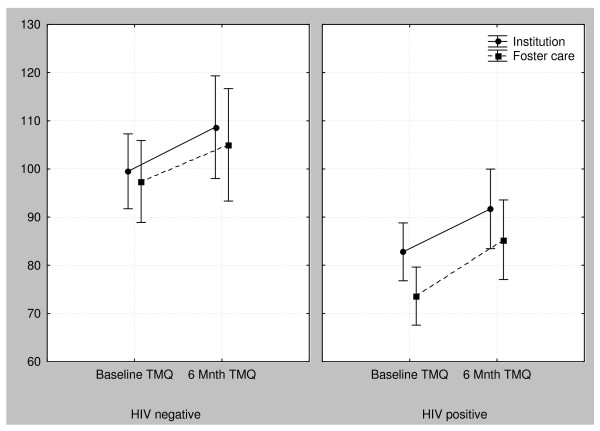
**Comparison of the change in Total Motor Quotients (TMQ)**. Comparison of the TMQ at baseline and six months of children with and without HIV, living in institutions and in foster care.

The GMQ, FMQ and TMQ were all normally distributed (Kolmorogov Smirnoff test -in each case p >.20) and therefore parametric tests were used. No correlation was found between age and TMQ (r = .03, p = .833) which implies that the older children did not perform relatively better than the younger children. The mean scores of the four groups of children indicate that children in foster care generally performed worse than those in institutions and children with HIV generally performed worse than children who were HIV negative (Figure 1). The children with HIV performed significantly worse than did the other children on all sections of the test (Table 3). The difference was greatest in the GMQ where the mean difference was 18.3. The differences in means were 12.7 and 16.0 for the FMQ and TMQ respectively. Children with HIV performed better with regard to the FMQ than the GMQ, but not significantly so (p = .08). Children with HIV in institutions performed significantly better in the FMQ (p < .001) and TMQ (p = .02) than those in foster care. This difference was not noted in children without HIV but in all cases the institutionalized children scored higher.

**Table 3:** Comparison of motor scores between groups

	*Mean (SD)*	*Mean (SD)*	*t-value*	*df*	*P*
**HIV status**	**Positive (N = 23)**	**Negative (N = 21)**			

GMQ	77.9 (11.5)	96.1 (11.5)	-5.3	42	<.001

FMQ	83.8(13.2)	96.6 (13.80	-3.1	42	<.001

TMQ	79.3(10.2)	95.3 (11.80	-4.8	42	<.001

**Residential setting**	**Foster (N = 19)**	**Institute (N = 25)**			

GMQ	75.6(14.3)	79.62(9.10	-0.82	21	.42

FMQ	75.1(12.80	90.54(9.2)	-3.36	21	<.001

TMQ	73.6(10.7)	83.69(7.7)	-2.65	21	.02

Comparison of the Gross Motor, Fine Motor and Total Motor Scores between groups (N = 44)

The regression analysis model that best fitted the data included status and residence as the other variables did not contribute significantly to the predicted value. HIV status and Residence were found to be predictive of TMQ and the model accounted for 42% of the variance (Adjusted R^2 ^= .42) (Table 4). Children in institutions were predicted to score 8.3 points higher than children in foster care. Similarly children without HIV were predicted to score 16 points more than their counterparts with HIV.

**Table 4:** Factors that predicted the baseline Total Motor Quotient score

	B	**Std.Err**.	t(41)	p-level
**Intercept**	98.9	2.616	37.81	<.001

**Status (HIV+)**	-16.0	3.106	-5.14	<.001

**Residence (Foster Home)**	-8.3	3.132	-2.66	.011

Results of multiple regression analysis with TMQ as dependent variable.(n = 44).

### Comparison of scores at 6 months

Three tests were incomplete as children were uncooperative and despite considerable effort to engage the children, testing could not be done. Seven children were discharged from institutions. In total 33 children were tested twice, 21 of whom were HIV positive.

The baseline TMQ was predictive of the six month TMQ (r = .76, p < .001) and all groups of children significantly improved over the six months (p < .001) in GMQ,FMQ, and TMQ. Forward stepwise analysis with the six month TMQ score as dependent measure and the same variables as above indicated that for all children no variables were predictive of TMQ. For the HIV positive group, residence and time on ARVT were included in the final model (Intercept = 83, adjusted R^2 ^= 0.19). Residence in foster care was found to be predictive of a worse outcome (B = -13.4, p = .048)) but time on ARVs was not (B = 0.4, p = .061).

## Discussion

The children in the four sub-samples were very similar in age and gender and the differences found are likely to be as a result of either their HIV status or their residential setting. Very few children had the experience of living with their natural parents and families before institutionalisation or fostering. A large percentage of children in foster care were cared for by older members of the various communities. The limited number of people prepared to foster children, the increase in the number of single parent households due to the impact of the migrant labour system and parents leaving to work or parents having difficulty coping with raising their children [16] might explain this finding. The phenomenon of elderly persons caring for young children is common in South Africa where the progression and spread of the HIV/AIDS epidemic has led to an increase in the grandparent headed households [17] and this is predicted to rise.

As anticipated, children with HIV performed at a lower motor level than their peers in both settings, a finding consistent with the literature [6,18,19]. Although many of the children reported serious illnesses at some stage of their lives prior to testing, none of the children experienced any serious illness at the time of testing and none of them were ill as a result of opportunistic infections for the duration of the study. The improved health status of the infected children is likely to have been due to the administration of ARVT to all the children and none of them had been on treatment for less than five months. A study in Uganda, reported that treatment on ARVT for six months or longer resulted in increased longevity and an improved health status [20]. The fact that the health status of all the children was good implies that the decreased scores for their development obtained on the PDMS II were unlikely to be due to current illness and/or recent hospital admissions.

FMQ was advanced in all institutionalised children. Fine motor activity depends on the ability to perform precise movement co-ordination and includes elements of hand eye co-ordination and that of manual dexterity which required increased concentration [21]. As fine motor function is related to practice and opportunities to use the upper limb in skilled tasks, it might be that the children in institutions were subjected to more play stimulation than children raised by foster parents, who were often fostering several children. The presence of skilled personnel and a large number of volunteers working at the institutions might have afforded these children greater attention and stimulation.

In a study on the dual impact of institutionalisation and HIV on 64 children in the Ukraine, the increased opportunities for play and use of toys within a well resourced institutional setting compared to foster care were also noted [6]. In contrast to the current study, children who were in institutions, whatever their HIV status, performed worse than those in family care. However, in the Ukraine study, the children who were institutionalised seemed to have suffered from a greater degree of early deprivation than those staying with families and the families were their own biological families and this represents an important confounding variable. The result of a study comparing 94 children in institutions with 48 in foster care in Iraq Kurdistan was somewhat more equivocal. It concluded that there were more similarities than differences between the two samples, although the fostered children did show more improvement in activity scale, externalizing problem scores and posttraumatic stress disorder-related symptoms [8]. A major difference between the Iraq study and the current research is that the 84% of the fostered children had a relative as a formal caregiver, whereas in our study no children were cared for by family members. It might be that the relationship of the foster parents to the child may impact on the quality of care and degree of stimulation that the child receives.

A striking finding was that a significant improvement was noted in the performance of all participants from baseline to six months. Although the researcher did give advice to care-givers due to ethical considerations, it is unlikely that a single session of advice on infant stimulation could have resulted in such a large change. The improvement was not a function of maturation as no correlation between age and TMQ was found and scores on fine motor development have been found to remain stable over time, from two to five years of age [22,23]. Exposure to the test at baseline might have led to a learning effect which resulted in improved performance on the second test. However this is not likely as the tests were six months apart. The improvement could have been as a result of the initiatives described in the research setting to empower care-givers to provide more developmental stimulation to their children.

It needs to be stressed that the current study did not examine the emotional well being of the children or their cognitive development. The results are not incompatible with the suggestion that long term fostering offers children security, a loving family environment and a close substitute of parental relationships while institutional care is thought to provide unstable caregiver relationships, lack of a sense of belonging and children may be missing out on the family unit [24]. However, this is not necessarily so in every case. There has been concern expressed over 'voluntourism' in which the impact of having international volunteers working in institutions for relatively short periods of time is being questioned in terms of the rejection that children may feel when the volunteer returns home [25]. There is a clear need for increased stimulation of these children both within the residential care and the foster care setting and it would be a pity if all resources were not mobilised. If it is the case that children in residential care are disadvantaged, well meaning and suitably trained volunteers could also interact with foster parents and provide additional support to children within the community.

### Limitations

The sample size was smaller than planned but there were still significant differences noted between groups and settings. Attrition is one of the greatest problems associated with longitudinal studies and the attrition in this study was about 20%, which is high, but not as high as the 58% attrition at six months reported in a similar study in Cape Town on younger children with HIV cared for by their mothers [13].

## Conclusions

The results are encouraging in that children seem to be well cared in both settings and the children seem to be accessing regular ARVT. This is evident in the lack of hospitalisations and opportunistic diseases reported. However, children with HIV were significantly delayed compared to their HIV negative counterparts. At the time of this study, children in South Africa were only put on ARVT if their CD4 counts were less than 15-20% of normal [26]. As this regimen did not appear to result in normalised motor performance during the tested six month window, the possibility that the motor developmental delay exhibited by these children will persist needs to be researched further.

Children in foster care, and particularly those with HIV, were found to perform worse in the area of fine motor skills. The difference decreased over the course of the study, as the level of stimulation to the foster care children increased, which implies that performance on the PDMS II is influenced by changes in the environment. This might indicate that the fostered children, especially those with HIV, need more stimulation and that this group needs to be targeted in future. This need is also likely to be evident amongst children with HIV living with their natural parents, or being cared for by relatives as the socio-economic setting is most probably similar [27].

Whereas, the researchers are loathe to conclude on the basis of this research that residential care is the preferred option, the assumption that foster care is superior to institutionalized care in every case needs to be revisited, particularly in under resourced areas. Further research on larger cohorts, including all aspects of cognitive, emotional and not only motor development should be undertaken to validate the findings of this exploratory study.

## Competing interests

The authors declare that they have no competing interests.

## Authors' contributions

JJ conceptualised the project and was the principal author of the paper, ND did data collection and contributed to the paper, GF monitored the quality of data collection and contributed to writing the paper. All authors read and approved the final manuscript.

## Pre-publication history

The pre-publication history for this paper can be accessed here:

http://www.biomedcentral.com/1471-2431/11/11/prepub
